# Complete paternally inherited mitogenomes of two freshwater mussels *Unio pictorum* and *Sinanodonta woodiana* (Bivalvia: Unionidae)

**DOI:** 10.7717/peerj.5573

**Published:** 2018-09-11

**Authors:** Artur Burzyński, Marianna Soroka

**Affiliations:** 1 Department of Genetics and Marine Biotechnology, Institute of Oceanology Polish Academy of Sciences, Sopot, Poland; 2 University of Szczecin, Faculty of Biology, Department of Genetics, Szczecin, Poland

**Keywords:** DUI, mtDNA, Mitogenomics, Phylogeny, Molecular clock

## Abstract

Freshwater bivalves from the family Unionidae usually have two very divergent mitogenomes, inherited according to the doubly uniparental model. The early divergence of these two mitogenomic lineages gives a unique opportunity to use two mitogenomic data sets in a single phylogenetic context. However, the number of complete sequences of the maternally inherited mitogenomes of these animals available in GenBank greatly exceeds that of the paternally inherited mitogenomes. This is a problem for phylogenetic reconstruction because it limits the use of both mitogenomic data sets. Moreover, since long branch attraction phenomenon can bias reconstructions if only a few but highly divergent taxa are considered, the shortage of the faster evolving paternally inherited mitogenome sequences is a real problem. Here we provide, for the first time, complete sequences of the M mitogenomes sampled from Polish populations of two species: native *Unio pictorum* and invasive *Sinanodonta woodiana*. It increases the available set of mitogenomic pairs to 18 species per family, and allows unambiguous reconstruction of phylogenetic relationships among them. The reconstructions based on M and F mitogenomes which were separated for many millions of years, and subject to differing evolutionary dynamics, are fully congruent.

## Introduction

Freshwater mussels from the order Unionoida are known for the widespread occurrence of doubly uniparental inheritance (DUI) of mitochondria (Walker et al., 2006). Under DUI all males are heteroplasmic and pass their mitochondrial DNA to male offspring whereas females pass their mitochondria to all their offspring (Skibinski, Gallagher & Beynon, 1994; Zouros et al., 1994). Consequently, the two independent mitochondrial lineages exist. In freshwater mussels (order Unionoida) the divergence between the two lineages is extreme because their emergence predates the family level radiation (Hoeh, Stewart & Guttman, 2002). The maternal (F) and paternal (M) mitogenomic lineages in Unionidae, the best studied of families from this order, differ slightly in genome organization but the genomes are fairly conservative structure-wise within each lineage, with only one rearrangement leading to alternative F gene order in one group of Unionidae (Breton et al., 2009). The two mitogenomes accumulated several unique gender-specific features, in the form of additional ORFs (Milani et al., 2013; Mitchell et al., 2016a) as well as extensions of the existing *cox2* CDS (Chapman et al., 2008). These features seem to be associated with DUI and sex determination systems because in species reverting to hermaphroditism both DUI and these peculiar mitogenomic features are eventually lost (Soroka & Burzyński, 2017).

Mitogenomic data are frequently used to reconstruct and date phylogenies (Mitchell et al., 2016b; Burzyński et al., 2017; Kieren et al., 2018; Krzeminska et al., 2018). The existence of two parallel mitochondrial lineages provides a unique opportunity to test the limits of such methodology (Burzyński et al., 2017). This is important in the context of critical re-evaluation of the usability of mitochondrial markers in resolving phylogenies (Wallis et al., 2017).

The first complete mitogenome of a unionid bivalve, *Lampsilis ornata*, was published more than 15 years ago (Serb & Lydeard, 2003) and the number of complete mitogenome sequences available in public databases is rising rapidly ever since. There are currently complete sequences of F-type mitogenomes of at least 24 species from two major subfamilies: Unionini and Anodontini alone in GenBank (Soroka & Burzyński, 2017). However, there are much fewer M-type mitogenomes present in GenBank, with only nine members of these two subfamilies represented, and a total of 17 M-type mitogenomes available for the whole Unionidae family (Table 1). Notably, two M-type mitogenomes of species from the sister family Margaritiferidae have been published recently (Lopes-Lima et al., 2017a; Guerra et al., 2017).

**Table 1:** Latin names, GenBank accession numbers and literature references for all mitogenomes used in comparative analysis. Most of the M mitogenomes were referenced in the same publication as the F mitogenome from the same species, as indicated by “F” in the “Reference M” column. Some relevant mitogenomes present in GenBank do not have a formal publication. The two sources of *S. woodiana* mitogenomes are indicated by country code appended to the latin name.

Latin name	Accession F	Reference F	Accession M	Reference M
*Aculamprotula tortuosa* (Lea, 1865)	KC109779	Wang, Cao & Li (2013)	KC441487	–
*Anodonta anatina* (Linnaeus, 1758)	KF030964	Soroka & Burzyński (2015)	KF030963	Soroka & Burzyński (2016)
*Arconaia lanceolata* (Lea, 1856)	KJ144818	Wang, Guo & Li (2016)	KJ775864	–
*Lamprotula leaii* (Griffith & Pidgeon, 1833)	JQ691662	–	KC847114	–
*Margaritifera marocana* (Pallary, 1918)	KY131953	Lopes-Lima et al. (2017a)	KY131954	F
*Margaritifera monodonta* (Say, 1829)	KU873123	Guerra et al. (2017)	KU873124	F
*Potamilus alatus* (Say, 1817)	KU559011	Wen et al. (2017)	KU559010	F
*Potomida littoralis* (Cuvier, 1798)	KT247374	Froufe et al. (2016)	KT247375	F
*Pronodularia japanensis* (Lea, 1859)	AB055625	–	AB055624	–
*Pyganodon grandis* (Say, 1829)	FJ809754	Breton et al. (2009)	FJ809755	F
*Quadrula quadrula* (Rafinesque, 1820)	FJ809750	Breton et al. (2009)	FJ809751	F
*Sinanodonta woodiana* (Lea, 1834) CH	KM272949	Zhang et al. (2016)	KM434235	–
*Sinanodonta woodiana* (Lea, 1834) PL	HQ283346	Soroka (2010)	MH349359	This study
*Sinohyriopsis cumingii* (Lea, 1852)	KM393224	Wei et al. (2016)	KC150028	–
*Solenaia carinata* (Heude, 1877)	KC848654	Huang et al. (2013)	KC848655	F
*Unio crassus* Retzius, 1788	KY290447	Burzyński et al. (2017)	KY290450	F
*Unio delphinus* Spengler, 1793	KT326917	Fonseca et al. (2016)	KT326918	F
*Unio pictorum* (Linnaeus, 1758)	HM014133	Soroka & Burzyński (2010)	MH349358	This study
*Unio tumidus* Retzius, 1788	KY021076	Soroka & Burzyński (2018)	KY021075	F
*Utterbackia peninsularis* Bogan & Hoeh, 1995	HM856636	Breton et al. (2011)	HM856635	F
*Venustaconcha ellipsiformis* (Conrad, 1836)	FJ809753	Breton et al. (2009)	FJ809752	F

It has been suggested that sparse and uneven taxon sampling biases the phylogenetic reconstructions, necessitating the need for more M mitogenomic data. To fill this gap, and check the usability of double-mitogenome approach in phylogenies, we sequenced complete M-type mitogenomes of two freshwater unionid species from Poland: the invasive *Sinanodonta woodiana* from Anodontini and the native *Unio pictorum* from Unionini. We selected two species for which the F-type mitogenomic data were already published (Table 1). Moreover, for *S. woodiana* there was also one GenBank record describing the complete M-type from the native range of the species, without formal publication.

## Methods

Mussels were sampled from the Oder River, 25 km south of the city Szczecin (in the north-western part of Poland, 53.2123N 14.4673E) in 2006. The bivalves were collected in May, June and July when their sex could be reliably identified by microscopic examination of their mature gonads. Tissue samples were immediately collected and processed for DNA isolation. Two males were selected at random from each species for full mitogenome sequencing. DNA was extracted from gonads using established methodology (Soroka & Burzyński, 2010). Material used in this study has been kept in 70% ethanol at the repository of the Department of Genetics, University of Szczecin with voucher numbers: AW5 and AW10 (*S. woodiana*), UP149 and UP232 (*U. pictorum*). Long Range Polymerase Chain Reaction (LR-PCR) primers were designed and used to amplify the complete M and F mitogenomes in two overlapping fragments, followed by primer walking approach (Table S1). Individual PCR products were then sequenced using ABI Big Dye Terminator technology in Macrogen (South Korea). Raw sequencing reads were trimmed and assembled using Staden package (Bonfield, Smith & Staden, 1995).

Complete mitochondrial genomes were annotated as described previously (Zbawicka, Burzyński & Wenne, 2007), using CRITICA (Badger & Olsen, 1999), GLIMMER (Delcher et al., 1999), BLAST (Altschul et al., 1990; Zhang & Madden, 1997), Wise2 (Birney, Clamp & Durbin, 2004), HMMER (Wheeler & Eddy, 2013), and ARWEN (Laslett & Canbäck, 2008). Annotated sequences have been deposited in GenBank under the following accession numbers: MH349356 (AW5), MH349357 (UP232) MH349358 (UP149) and MH349359 (AW10). The annotations present in GenBank record KM434235 were inspected and adjusted to match those of MH349357 and MH349359, for consistency, using the same methodology.

MEGA7 (Kumar, Stecher & Tamura, 2016) was used to align sequences and calculate all diversity indices. Sliding window analysis of genetic diversity was done in DnaSP (Librado & Rozas, 2009). Circular maps and nucleotide composition analyses were produced in CGView (Stothard & Wishart, 2005). All other sequence manipulations, such as feature extraction and alignment concatenation, were performed in CLC Genomics Workbench version 9.5.4 (https://www.qiagenbioinformatics.com/).

A data set for phylogenetic reconstructions of relationships within Unionidae was selected based on the availability of the complete mitogenomic sequences from both lineages, belonging to Unionidae family and present in GenBank (*nr* database) at the time of this writing (February 2018). In addition to all such pairs of sequences from Unionidae, two species from Margaritiferidae family were also used to serve as an outgroup. These two families are generally considered to have a sister relationship (Walker et al., 2006; Bogan & Roe, 2008). The details of species names and accession numbers, along with the relevant references, are given in Table 1. All taxonomic names were verified and adjusted according to MUSSELp (2018) (http://mussel-project.uwsp.edu/) database. To overcome the problems associated with potential shifts of mutational pressure due to genomic rearrangements as well as with saturation effects, the analysis should be performed in amino acid space. However, as previously noted (Burzyński et al., 2017), some database sequences do contain suspicious frame shifts leading to unreliable amino acid alignments and biased or plainly wrong reconstructions. To overcome this problem the following procedure was applied. First, all protein coding genes were extracted and translated, following the existing CDS annotations. Then the alignments were produced in both amino acid and nucleotide space and inspected, for each gene and lineage separately. Sequences, for which the nucleotide alignment was inconsistent with the amino acid alignment were deemed suspicious and were removed from the alignments. The curated alignment was then used to produce hmm alignment profile (Finn, Clements & Eddy, 2011), of each protein in both lineages (26 hmm profiles). Finally, these profiles were used to recover protein alignments directly from raw sequence data using *genewise* (Birney, Clamp & Durbin, 2004). Amino acid alignments were concatenated and used in all subsequent analyses (protein alignments). The rRNA genes were extracted separately and used directly after alignment and concatenation (rDNA alignments). The lengths of all alignments were: 3,907 aa (F), 4,240 aa (M), 2,273 bp (F) and 2,389 bp (M). However, since the gap columns are not used in reconstructions, the alignments were effectively shorter: 3,636 aa (F), 3,524 aa (M), 1,738 bp (F) and 1,694 bp (M), for protein and rDNA alignments of F and M lineages, respectively. The apparently high proportion of gaps in the M protein alignment is caused solely by the unusually short *cox2* extension in one species: *Potamilus alatus* (Wen et al., 2017), and is not indicative of any alignment problems. All four alignments are available as Supplemental Information 1.

Phylogenetic reconstructions by Bayesian Inference (BI) were done in BEAST2 (Bouckaert et al., 2014), as described previously (Burzyński et al., 2017; Soroka & Burzyński, 2017). Input XML files for BEAST were prepared in BEAUTi. All the analyses were run in quadruplicates, for 10^7^ generations. The resulting log files were inspected in Tracer (Rambaud & Drummond, 2013) to verify the convergence of the runs. There was a good agreement between the repeats, hence all four log files as well as tree files were combined in *logcombiner*, after removal of 10% burn-in states. Each parameter has reached effective sample size (ESS) of more than 300, in the combined log file. The consensus tree was obtained from the concatenated treefile in *treeannotator.* There were three such analyses: one using only the M-type data, one using only the F-type data, and one using the data obtained from both mitogenomes in a single reconstruction. Each alignment was added as a separate partition, with individually selected model of substitutions, but with the same tree for all partitions. The optimal model of substitutions was selected, following the recommended Path Sampling procedure (Baele et al., 2012; Baele et al., 2013). Consistently, mtRev model was selected for protein partitions and GTR model for rDNA partitions, both with gamma-distributed heterogeneities of rates. Relaxed, uncorrelated lognormal clock was used following previous recommendations (Burzyński et al., 2017), and the Yule tree prior was assumed in all analyses. The trees were visualized in FigTree (Rambaut, 2009). To allow the comparison of the obtained topologies as well as node heights, all trees were arbitrary normalized to the most recent common ancestor (MRCA) of the family Unionidae.

Phylogenetic reconstructions were also performed under Maximum Likelihood (ML) framework, as implemented in IQ-TREE (Nguyen et al., 2015). First, the optimal model was selected for each individual alignment separately, using *modelfinder* (Kalyaanamoorthy et al., 2017). The following models were chosen by BIC criterion: for protein alignments mtVer+F+R5 (F), mtMet+F+R4 (M) and for both rDNA alignments TIM2+F+I+G4. Then, the three reconstructions were performed, using partitioned data consisted of either two M-type, two F-type or all four alignments in a single reconstruction (Chernomor, Haeseler & Minh, 2016). Finally, to access the stability of the obtained topology, *ultrafast* bootstrap procedure was used, with 1,000 replicates (Minh, Nguyen & Haeseler, 2013).

Separate analysis was done on a portion of M-type mitogenome containing m-ORF to obtain estimates of relative divergence of the two copies of m-ORF apparently present in *S. woodiana* mitogenome. Since these sequences evolve exceptionally quickly and can only serve comparisons at subfamily level at best (Mitchell et al., 2016a), it is impossible to produce reliable amino acid alignment for them. To overcome this difficulty, m-ORF annotations were extracted from the five closest relatives (FJ809751, KF030963, HM856635, KM434235 and MH349359) and aligned in nucleotide space. This short alignment (seven sequences, 429 positions) was then used in BEAST to reconstruct the phylogeny of duplication, assuming GTR model of substitutions, with gamma-distributed rates and lognormal relaxed clock. Other parameters and run conditions were the same as for the main phylogenetic reconstruction.

## Results

**Figure 1:**
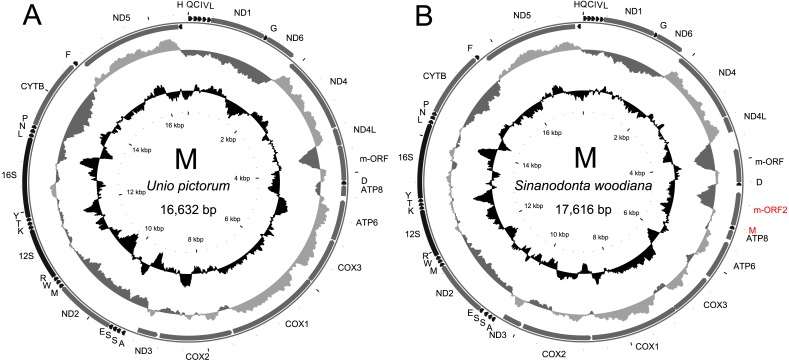
Genetic map of the sequenced M-type mitogenomes. The mitogenome of *U. pictorum* (A) is structurally very similar to all M-type mitogenomes from this family published to date. The mitogenome of *S. woodiana* (B) is more exceptional because of the two additional features labeled in red. All protein coding genes are labeled by the names of the encoded proteins, two *rRNA* genes are labeled 16S and 12S for large and small subunit, respectively. The *trn* genes are labeled by the one letter code of the respective amino acid. Direction of transcription is indicated by the position and direction of the arrows, with clockwise transcribed genes on the outside and anticlockwise transcribed genes on the inside of the circle representing the genome. The two inner circles represent local compositional bias, calculated in a 300 bp long window, in 25 bp long steps. The light gray parts of the first circle represent above average AT skew [(A–T)/(A+T)] while the dark gray parts of this circle represent below average AT skews. The inner, black circle represents local GC content. Both indices are calculated with the relation to mitogenome-wide averages which are, for *S.* woodiana: AT-skew -0.3166, GC content 0.3284, for *U. pictorum*: AT-skew -0.2828, GC-content 0.3396.

The two sequenced M mitogenomes of *U. pictorum* are almost identical, with only six substitutions along the whole 16,632 bp, therefore only one was used in all subsequent analyses (Fig. 1). These genomes are very similar both in structure and in sequence to other M mitogenomes from the compared data set. Particularly striking is the similarity to the M mitogenome of *U. delphinus,* with the nucleotide p-distance at the barcoding *cox1* locus of only 0.04.

**Figure 2:**
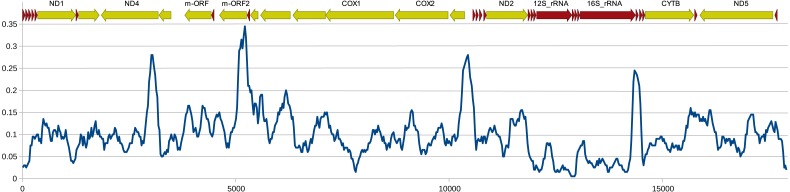
Genetic distance between Chinese and Polish M-type mitogenomes of *S. woodiana*. Uncorrected nucleotide p-distance calculated in a sliding window of 300 bp along the alignment of the two M-type mitogenomes of *S. woodiana*, one sampled in Poland, the other in China. Schematic annotation is showed above the plot, with the major genes labeled.

**Figure 3:**
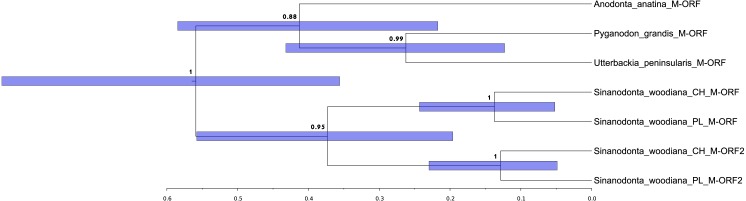
Relationships between duplicated m-ORF sequences. Phylogenetic reconstruction of the relationships between the m-ORFs of the seven closely related sequences derived from four species. The tree is based on Bayesian inference, the nucleotide alignment contained 429 bp. Posterior probability is shown at all nodes, as well as 95% confidence interval of node height (blue bars).

**Figure 4:**
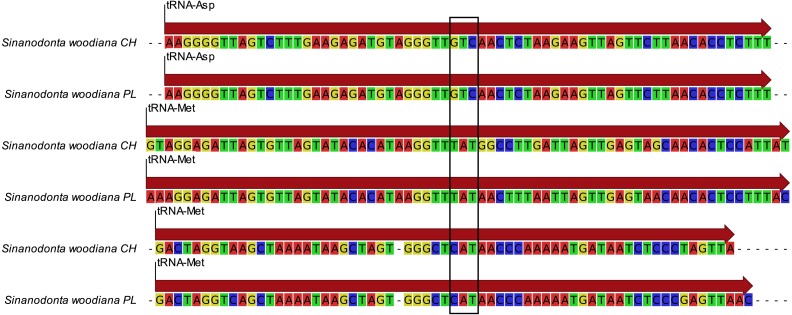
Comparison of the new *trn.* genes. Alignment of the three *trn* genes from the two M-type mitogenomes of *S. woodiana*. The top two lines represent the regular *trnASP* gene, the two bottom lines represent the typical *trnMET* gene. The two lines in the middle represent the novel *trn* gene. Sequences extracted from both M-type mitogenomes are shown, one from Poland (PL), one from China (CH). The position of anticodon sequence is boxed.

The structure and pattern of divergence for the second set of sequenced mitogenomes, the M mitogenomes of *S. woodiana* are different. Again, the two sequenced mitogenomes were very close to each other (only 22 substitutions, 17,616 bp) allowing the use of a single representative in all subsequent analyses. However, the BLAST search in *nr* database revealed the presence of unpublished mitogenome annotated as M mitogenome of the same species, sampled in China (Table 1). The M mitogenomes of *S. woodiana* sampled in China and sampled in Poland clearly belong to very distant clades because they differ in sequence substantially (average nucleotide p-distance 0.097). The spread of polymorphisms along sequence alignment (Fig. 2) shows fairly typical pattern, with less divergence in rDNA loci but also with several short anomalously divergent regions. However, despite these differences, the mitogenomes are structurally identical and unique among the mitogenomes published to date (Fig. 1). Instead of one supranumerary m-ORF they possess two copies of this gene and one additional *trnMET*. The two copies of m-ORF are quite distinct, clearly their emergence predates the divergence of the two M-clades in *S. woodiana*, as suggested by the phylogenetic reconstruction involving their closest relatives (Fig. 3). The inspection of the *trnMET* suggests that it is utilizing TAT anticodon but otherwise resembles the *trnASP* rather than the canonical *trnMET* serving the start codons (Fig. 4).

**Figure 5:**
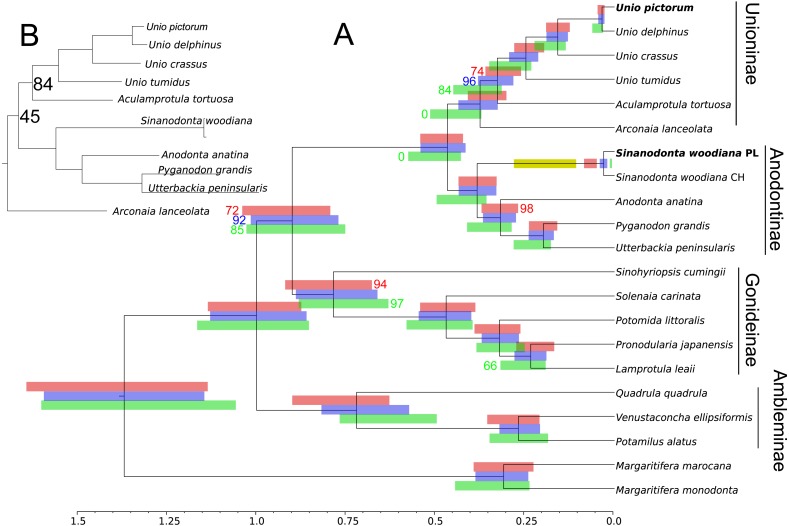
Phylogenetic reconstruction of relationships within Unionidae. Phylogenetic reconstruction of relationships within Unionidae based on the concatenated amino acid alignment of all 13 mitochondrially encoded proteins and nucleotide alignment of the two rRNA genes, from both M and F type mitogenomes (A). Six separate reconstructions were performed: three under BI and three under ML framework, separately for the F and M data sets as well as jointly for both data sets. The presented tree is a result of BI under joined data set but the topology of all three trees obtained under BI was the same. All nodes have posterior probability of 1.0. The 95% CI on node heights is shown as blue bars at nodes for the joined M+F analysis, as red bars above nodes for the analysis based on the M data set and as green bars below nodes for the analysis based on the F data set. The topology obtained under ML framework for M and for joined M+F data sets was the same, with 100% bootstrap support for most of the nodes. The nodes with less than 100% bootstrap support are indicated by the actual percentage numbers next to the CI bars, in the color corresponding to the used data set (red for M, green for F, and blue for M+F ). The two “zero” values in green indicate that the topology recovered by ML under the F data set was different, placing *A. lanceolata* outside the Unioninae clade with poor support, so that these nodes were not present. Relevant fragment of this alternative tree is also shown (B), again only nodes with less than 100% support are labeled. The yellow bar is the 95% CI on the MRCA of the apparent duplication of m-ORF within the *S. woodiana* lineage, transferred from Fig. 3.

Phylogenetic reconstruction (Fig. 5) revealed good agreement between the M and F data sets. The relationships between all taxa were resolved consistently in all three BI reconstructions and, after normalization to the Unionidae MRCA node, the node heights were also reasonably congruent. The two divergent haplogroups of *S. woodiana* present in the M data set clearly did not have the counterparts in the F data set but apart from this exceptional case all node heights were estimated more reliably using two data sets simultaneously. ML reconstructions yielded the same topology for M and for joined data sets, with good support for the majority of nodes and notable increase in support in the joined analysis. However, when only the F data set was used, the alternative topology was recovered, placing *Arconaia lanceolata* at the root of Unioninae + Anodontinae clade. The support for this topology was poor and all the other parts of the tree were congruent with the remaining analyses.

## Discussion

Presence of two divergent mitogenomes following the gender-specific distribution in Unionoida gives the opportunity to analyse structural evolution of the mitogenomes along a long evolutionary timescale. Relatively few structural changes has been revealed in this group so far (Lopes-Lima et al., 2017a). In particular no changes in gene repertoire has been noticed to date. Here we report, for the first time a case of major CDS duplication involving the m-ORF of *S. woodiana.* Moreover, the single duplication event covered also the adjacent *trn* gene, leading to the emergence of a new *trn*, with TAT anticodon. It is more parsimonious to assume that this new *trnMET* originated from the duplicated *trnASP* by the change of specificity than by independent “jump” of *trnMET* which would also require the change in anticodon sequence (Fig. 4). A *trnMET* gene of the same specificity is present in Mytilidae (Boore, Medina & Rosenberg, 2004), but has not been reported in freshwater mussels so far. This shows, that the typical small-scale mitogenomic rearrangements may be more common in Unionoida than could have been expected based on the limited data set currently available. The origin of this duplication is uncertain, but based on the phylogenetic reconstructions (Figs. 3 and  5), it seems to be relatively old, possibly predating species level radiation within the genus. Further mitogenomic data from other members of the genus *Sinanodonta* will no doubt allow more precise analysis of this phenomenon.

Mitochondrial markers were traditionally used in phylogeny reconstructions due to their favorable properties of no recombination and clonal inheritance (Avise, 1986). Early attempts to apply this approach to Unionidae, using short fragment (650 bp) of F-type *cox1* sequences produced poorly resolved tree, suggesting non-monophyletic Unionidae (Bogan & Hoeh, 2000). Inclusion of more taxa in later analyses allowed for recognition of three well-supported clades within Unionidae (Unioninae, Anodontinae and Ambleminae) as well as confirmation of Margaritiferidae as a sister group to monophyletic Unionidae (Graf & Ó Foighil, 2000). However, this marker alone was not sufficient to resolve the internal relationships within Unionidae with any certainty. Increasing the length of mitochondrial sequences by adding 753 bp of *nd1* and 315 bp of *16S* resulted in a limited increase of support for some nodes but the relationship between the major clades remained unsolved (Campbell et al., 2005). The addition of a nuclear rDNA marker (473 bp of *28S*) along with several morphological characters was also not helpful (Graf & Cummings, 2006). More recent study using similar *cox1*, *16S,* and *28S* markers (alignment length 1,900 bp) produced similarly inconclusive reconstruction (Pfeiffer & Graf, 2015). Only recently the complete mitogenomic data has been applied to phylogeny reconstruction within Unionidae. The most straightforward analysis, with 12 protein coding genes aligned separately and concatenated did not result in conclusive resolution of all relationships (Fonseca et al., 2016; Froufe et al., 2016). The support for monophyletic Anodontinae + Unioninae was overwhelming in both M-type and F-type clades. However, the position of Gonideinae on the tree was uncertain and inconsistent between F-type and M-type clades. More recent comprehensive attempts to solve the phylogeny of Unionidae used more sophisticated methods and much better sampling, but were still based on a very limited set of characters: the same *cox1, 28S* and *16S* markers (Lopes-Lima et al., 2017b; Bolotov et al., 2017). Both analyses agree that Ambleminae form a sister relationship with Gonideinae. The same conclusion was supported by our recent mitogenomic-based analysis (Burzyński et al., 2017). The phylogeny reconstructed here (Fig. 5) suggests different relationship of the three major clades: places Ambleminae as a basal clade and Gonideinae as a sister group to Anodontinae + Unioninae. Similar topology was recovered in the most recent reconstruction (Lopes-Lima et al., 2017a), although with relatively poor support. The primary reason for this change is most likely the increased taxonomic sampling, particularly the inclusion of proper outgroup taxa from the sister Margaritiferidae family. The increased support for the problematic nodes is most likely caused by the retention of more informative characters in separate M and F alignments, demonstrating that the presented approach is more efficient. In addition to the proper solution of the relationship between the three major clades, the presented reconstruction places the species *Arconaia lanceolata* on the tree closer to the Unioninae than to the Anodontinae clade. This is also in conflict with most of the previous analyses (Lopes-Lima et al., 2017b), in agreement with the F-type clade presented by (Lopes-Lima et al., 2017a), but with much better support. The small remaining uncertainty regarding the placement of Gonideinae, apparent in the ML tree, is understandable, given the fact that this group shares one particular rearrangement within their F type mitogenome (Breton et al., 2009). Such rearrangements can lead to biased mutational pressure and consequently biased reconstruction. Moreover, the mitogenomic data are still lacking for several Unionidae subfamilies (Lopes-Lima et al., 2017b) and it is likely that when they are available the remaining uncertainty will disappear.

The presence of two apparently very divergent mitochondrial clades in a single species, as noted for *S. woodiana*, is frequently interpreted as indicative of cryptic speciation event. In fact similar interpretation was recently given to explain patterns of deep polymorphisms noted in barcoding *cox1* locus in *S. woodiana* populations worldwide (Bolotov et al., 2016; Bespalaya et al., 2018). This view is probable if one looks at comparable divergence between the two closely related *Unio* species (*U. pictorum* and *U. delphinus*): their separation on the tree (Fig. 5) is comparable to the one observed in *S. woodiana* and yet their status as separate species is supported by all recent phylogeographic analyses (Prie & Puillandre, 2014; Araujo et al., 2018). However, for *S. woodiana* the conclusion must not be straightforward. The fact that the two very divergent M clades are present in the set of populations where only a single F clade is present (PL and CH) argues strongly against such interpretation in this case. It may well be that a complex pattern of incomplete lineage sorting and/or hybridization events makes any classic barcoding attempts, that is species inferences based on F mtDNA *cox1* sequences, questionable in this case. Clearly more data are needed to explain this pattern, including the M mitochondrial markers along with markers based on nuclear loci.

## Conclusions

The presented mitogenomic data are a valuable resource for further phylogenetic and population genetic studies.

The presented novel approach to phylogenetic reconstructions using mitogenomic data should help to overcome methodological problems, particularly in solving and dating deeper phylogenies.

##  Supplemental Information

10.7717/peerj.5573/supp-1 Supplemental Information 1 Sequence alignments and input files for phylogenetic analysis Each file represents the alignment of particular set of loci in fasta format or in xml format suitable for direct use in BEAST.

10.7717/peerj.5573/supp-2 Table S1 List of primer sequences Primers used in PCR and sequencing of *U. pictorum* (U) and *S. woodiana* (A) M-type mitogenomes.

## Additional Information and Declarations

### DNA Deposition

The following information was supplied regarding the deposition of DNA sequences: The mitogenomes described here are accessible via GenBank accession numbers: MH349356 (AW5), MH349357 (UP232) MH349358 (UP149) and MH349359 (AW10).

### Data Availability

The following information was supplied regarding data availability: The raw data are provided in the Supplemental Files.
