# Supplementary material for: Complete paternally inherited mitogenomes of two freshwater mussels Unio pictorum and Sinanodonta woodiana (Bivalvia: Unionidae)
Source: PeerJ. 2018 Sep 11;6:e5573. doi: 10.7717/peerj.5573 (PMC6138038; doi:10.7717/peerj.5573)
Supplement: Table S1 — Primers used in PCR and sequencing of U. pictorum (U) and S. woodiana (A) M-type mitogenomes. [file peerj-06-5573-s002.pdf]

Supplementary Table S1: Primers used in PCR and sequencing of *U. pictorum* (U) and *S. woodiana* (A) M-type mitogenomes.

Name	Sequence	Specificity
AA106_R15	TTCGTGGATTATCGAATTAAG	A
M.aa.02	TCTTTGGGTCCTTTCGTACAA	UA
M.aa.06	GATGAACTATTTATCCGCCTTT	A
M.at8.01	TTAAATGCCTCAATTAAGTCCT	A
M.aw.01	TTATGAGCCCACTAGCTTATTTT	A
M.aw.01a	GCAAAAGGAGGTATTCC	A
M.aw.01b	TTAAGGTGTAAATAGCGAAGTAAAATAA	A
M.aw.02	AAACAGGTATAAAAGAGTGATTAACCC	A
M.aw.02a	AAGAGTGATTAACCCCG	A
M.aw.03	GGTTTGATAGCAAATGAACAGCTATATTATTC	A
M.aw.03a	AACAGGCGGTAGTAAATGAGGTG	A
M.aw.03b	GCGGTAGTAAATGAGGTGAATGTG	A
M.aw.04a	TTTCCTACTTCTTACATGATTGGGTC	A
M.aw.04b	GGGTCAAATACCAAGAGAAACTCC	A
M.aw.05	CTCCTAGTATTTCTATTCAACCCGC	A
M.aw.06	GATGGACTATTTATCCGCCTTTG	A
M.aw.07	TTCATTTAGCAAGTGTTTCATCAA	A
M.aw.08	CGAATGACATGACTCTACCCAA	A
M.aw.1d	ATAGCATGATCGTATAGGCATTG	A
M.aw.1e	TGTTAATTGACTGTAGGGAGTTACC	A
M.aw.LR.102	TCCTTTGTTTGTTTGATCTGTTTTGTG	A
M.aw.LR.202	GCAGGCAGTCAAACAGAAAAAGGTA	A
M.aw.Z1	CCAATGGATTATTTTGTCTACAGC	A
M.aw.Z2	TGTAGGTAGAGTAAACGATTATGGTTC	A
M.co2.01	TATCGTATATTGGAGGTAGATAATCG	A
M.co2.02	ATTTGACCTCAAAAGCTCATCTT	U
M.co3.01	TAAATGCGTAGTCCTTTTCATTT	U
M.co3.04	TGGAGTTAGTGTTACTTGATGTCA	U
M.co3.05	ACTTGTGTGTGTATTGATGAGGTGG	A
M.co3.06	TGTATTGATGAGGAGGGAGG	U
M.for.02	AACAGCACGAAACAAACCA	U
M.for.04	GGGTGGTTATAGAGGATGGTAA	A

M.glu.01	TACACTAAGCTAACAGTGCTGAGAA	U
M.lrn.01	AGCRAAATACCTATCGAGCCACTTG	U
M.lrn.02	CCTTAGAGCCCCTAGAAATAGC	U
M.nd1.03	GGTTAGGATTATTGGAATTTCG	U
M.nd4.01	ATATACAAAACCACTTCACCACAA	U
M.nd4.02	TGGAAACCTAAGTCTCCTACAAACG	A
M.nd4.04	CACCATAACTACAATTACCAATCC	U
M.nd4.06	AAGAAATAAACCTTATACAACCTAAAA	A
M.nd4.09	ACCGAGGCTTCAACATGAG	A
M.nd5.04	GTGAATGGAGATACTGGGTGG	U
M.nd5.05	TATACATAACTAATACTAGCCCTCAATAAA	U
M.nd5.06	ATACAATACCCAACAAATCCCA	U
M.nd6.02	AACTATAAAAATTATATAGGCATATCATGT	U
M.srn.01	ACCGCCACGTTCTTTAAGTTTGG	UA
M.up.01	TATGAGCCCACTAGCTTGGTT	U
M.up.01b	TGTGAATCTTAGTGGTTTTGAGTAG	U
M.up.02	AAATAGGCATAAAAGAGTGATTAATTCCGCATAACT	U
M.up.02b	GCTTCAACACAAATAGGCATAA	U
M.up.03	GGGATTTTATGCAATGAGCAGCTTTACTATTC	U
M.up.LR.101	CGTATGACTTTATTTGTATGATCTGTTTTGTG	U
M.ut.01	TTATGAGCCCACTAGCTTAGTTT	A
M.ut.02c	CATGGAAGAATGGTTCAAACA	U
M.ut.03b	TGAGGATTAGTGGGTTTGTCTTTAAG	U
M.ut.03c	TTATGGTGAGGATTAGTGGGTTTGT	U
M.ut.100	TGATCTGTTTTGTGTACGGC	UA
M.ut.Z2	TTTGGGTCCTTTCGTACAATAA	U
M.xxx.01	AAAAGATAGAATCCAACCTAGCTCT	UA
M.xxx.02	AAACTAGGCAACAGAGATAGTCAAT	UA
M.yyy.01	AAATACCTATCGAGCCACTTGATAGC	U
M121	TTGATATTTTTTCTTCATTAGACTATT	U
M132	TCAACTCGAATAATAACCCTTAAAG	U
M161	ATATCTCATTTTTTACTTCATCAAA	U
M212	GTCTTTAAGGGACAACCAGC	UA
M221	TAGCAGAAACTAATCGAACACC	A
M231	AAAACCACTTCACCACAA	U
M241	TACCCTCAACCGCAAATAA	U

UC.trn.04	TGCTTGGAAGGCAAT	U
UP.co1.02	CGGGGAAACGCTATATCAGGACA	U
UP.co1.03	CTTTATTTGTATGATCTGTTTTGTGTACGG	U
UP.co1.04	CTACTAATCATAAGGATATTGG	A
UP.lrn.03	TGCCTGTTTACCAAAAACATCG	A
UP.lrn.04	CACGCTCACGCTAACG	UA
UP.trn.01	TTTGGTAAAGTTGCAGTTTACAGTAA	U
UT.srn.01	TTATCGAATTAAGCCACAGG	UA
